# Evaluation of TiO_2_ Nanoparticle-Enhanced Palm and Soybean Biodiesel Blends for Emission Mitigation and Improved Combustion Efficiency

**DOI:** 10.3390/nano14191570

**Published:** 2024-09-28

**Authors:** Ramozon Khujamberdiev, Haeng Muk Cho

**Affiliations:** Department of Mechanical Engineering, Kongju National University, Cheonan 31080, Republic of Korea; khujamberdievramozon@gmail.com

**Keywords:** blended biodiesel, nanoparticle, titanium oxide, performance, emission, biofuel

## Abstract

The use of biodiesel as an alternative to conventional diesel fuels has gained significant attention due to its potential for reducing greenhouse gas emissions and improving energy sustainability. This study explores the impact of TiO_2_ nanoparticles on the emission characteristics and combustion efficiency of biodiesel blends in compression ignition (CI) engines. The fuels analyzed include diesel, SB20 (soybean biodiesel), SB20 + 50 TiO_2_ ppm, SB20 + 75 TiO_2_ ppm, PB20 (palm biodiesel), PB20 + 50 TiO_2_ ppm, and PB20 + 75 TiO_2_ ppm. Experiments were conducted under a consistent load of 50% across engine speeds ranging from 1000 to 1800 RPM. While TiO_2_ nanoparticles have been widely recognized for their ability to enhance biodiesel properties, limited research exists on their specific effects on soybean and palm biofuels. This study addresses these gaps by providing a comprehensive analysis of emissions, including NO_X_, CO, CO_2_, and HC, as well as exhaust gas temperature (EGT), across various engine speeds and nanoparticle concentrations. The results demonstrate that TiO_2_ nanoparticles lead to a reduction in CO emissions by up to 30% and a reduction in HC emissions by 21.5% at higher concentrations and engine speeds. However, this improvement in combustion efficiency is accompanied by a 15% increase in CO_2_ emissions, indicating more complete fuel oxidation. Additionally, NO_X_ emissions, which typically increase with engine speed, were mitigated by 20% with the addition of TiO_2_ nanoparticles. Exhaust gas temperatures (EGTs) were also lowered, indicating enhanced combustion stability. These findings highlight the potential of TiO_2_ nanoparticles to optimize biodiesel blends for improved environmental performance in CI engines.

## 1. Introduction

Biofuels, derived from organic materials such as plants and animal fats, present a renewable and sustainable alternative to fossil fuels. They have garnered attention due to their potential to reduce greenhouse gas emissions, enhance energy security, and promote environmental sustainability. Biodiesel, a major category of biofuels, can be produced from various feedstocks including palm oil and soybean oil. These biofuels can be directly used in existing diesel engines with minimal modifications, offering a significant reduction in carbon footprint compared to conventional fossil fuels [1,2,3]. Despite these advantages, biofuels face several challenges such as higher nitrogen oxide (NO_X_) emissions and lower oxidation stability compared to fossil fuels. These limitations require biofuel properties to be further optimized to meet emission standards and improve engine performance [4,5].

To address these challenges, the addition of nanoparticles to biofuels has shown promising results in enhancing fuel properties and engine performance. Nanoparticles, due to their small size and large surface area, act as effective catalysts and lubricants, leading to better combustion efficiency and reduced emissions. Nanoparticles, such as titanium dioxide (TiO_2_), cerium oxide, and aluminum oxide, have been extensively studied for their ability to improve fuel atomization, air–fuel mixing, and ignition delay reduction. These improvements contribute to higher engine efficiency, increased power output, and reduced harmful emissions such as NO_X_ and particulate matter [1,6].

Titanium dioxide (TiO_2_) nanoparticles have emerged as a particularly effective additive for enhancing biofuel properties. TiO_2_ nanoparticles improve the thermal and oxidative stability of biodiesel, leading to more efficient combustion and reduced engine wear. The catalytic properties of TiO_2_ nanoparticles facilitate oxidation reactions during combustion, promoting more complete fuel combustion and reducing the formation of unburned hydrocarbons (HC) and carbon deposits. Studies have shown that TiO_2_ nanoparticles can significantly enhance the combustion characteristics of biodiesel, resulting in improved fuel economy and lower emissions of pollutants such as carbon monoxide (CO), hydrocarbons (HC), and nitrogen oxides (NO_X_) [6,7].

The mechanism behind the improvement is largely attributed to the high surface area of TiO_2_ nanoparticles, which provides more active sites for catalytic reactions. This leads to better oxidation of fuel molecules and higher combustion efficiency. Additionally, TiO_2_ nanoparticles stabilize combustion temperatures, reducing NO_X_ formation, which is typically associated with high-temperature combustion [8,9]. Research indicates that the inclusion of TiO_2_ nanoparticles in biodiesel blends results in significant improvements in engine performance and emission characteristics. For instance, a study on Mahua biodiesel blended with TiO_2_ nanoparticles demonstrated enhanced combustion efficiency and reduced emissions at varying injection pressures [10]. Another study on tamarind oil biodiesel with TiO_2_ and alcoholic fuel additives showed similar benefits, with notable reductions in NO_X_ and particulate emissions [9].

Moreover, TiO_2_ nanoparticles have been used as catalysts in the synthesis of biodiesel, enhancing the conversion efficiency and quality of the biofuel. For instance, TiO_2_-catalyzed biodiesel production using *Carthamus tinctorius* L. oil showed significant improvements in yield and purity [11].

Comparative studies have highlighted the superior performance of TiO_2_ nanoparticle-enhanced biodiesel over traditional biodiesel and diesel blends. For example, the use of TiO_2_ nanoparticles in biodiesel from *Eichhornia crassipes* led to improved engine performance and lower emissions across various operating conditions [12]. Similarly, an experimental evaluation of *Semecarpus anacardium* biodiesel with TiO_2_ nanoparticles demonstrated enhanced stability and physicochemical properties, contributing to better engine performance [13].

The potential of TiO_2_ nanoparticles in biodiesel applications is further supported by their role in reducing soot and NO_X_ emissions in advanced engine technologies. The incorporation of TiO_2_ nanoparticles in biodiesel blends has shown to be effective in engines equipped with exhaust gas recirculation (EGR) systems, significantly lowering emissions without compromising engine performance [14].

### The Objectives of This Study

This study aims to investigate the emission and performance characteristics of TiO_2_ nanoparticles in palm and soybean biofuel in a compression ignition (CI) engine. The specific objectives are as follows:

1. To evaluate the influence of TiO_2_ nanoparticles on the reduction in harmful emissions (CO, HC, and NO_X_) and the enhancement in combustion efficiency in soybean and palm biodiesel blends.
2. To explore the potential of TiO_2_ nanoparticles in mitigating NO_X_ emissions and lowering exhaust gas temperatures (EGTs), particularly at higher engine speeds.

## 2. Literature Review

Titanium dioxide (TiO_2_) nanoparticles have been extensively studied for their potential to enhance the properties of biofuels. The integration of TiO_2_ nanoparticles into biodiesel has shown significant improvements in combustion efficiency, emission reduction, and engine performance, largely due to their catalytic properties, which enhance oxidation stability. Several studies highlight the role of TiO_2_ nanoparticles in acting as catalysts that improve the oxidation stability of biodiesel, which is crucial for efficient combustion and reduced emissions.

Several studies have highlighted the specific benefits of TiO_2_ nanoparticles in biodiesel blends. Rajak et al. (2020) discussed the benefits of using TiO_2_ nanoparticles in first-generation biodiesel blends, including palm and soybean oils, highlighting enhanced combustion characteristics and lower emissions [15]. Building on this, Lai et al. (2023) demonstrated that green fuel blends with TiO_2_ nanoparticles significantly optimized diesel engine performance and reduced pollutants [6]. These findings suggest that TiO_2_ nanoparticles not only improve the combustion process but also address the persistent issue of high NO_X_ emissions typically associated with biodiesel combustion, offering a more sustainable alternative to traditional diesel.

In comparative studies, TiO_2_ nanoparticles have demonstrated superior performance when used in combination with other additives. Pullagura et al. (2022) conducted a comparative study on the use of TiO_2_ nanoparticles and alcoholic fuel additives in biodiesel-diesel blends, showing that TiO_2_ nanoparticles improved the combustion efficiency and reduced NO_X_ emissions [9]. Bayindirli et al. (2023) also reported that TiO_2_ nanoparticles enhanced the thermophysical properties and combustion performance of biodiesel [2]. These studies underline the versatility of TiO_2_ nanoparticles in improving various aspects of biodiesel performance, from thermal stability to emission control.

Previous studies have established the baseline performance of palm and soybean biodiesel in compression ignition (CI) engines, laying the groundwork for exploring further enhancements with TiO_2_ nanoparticles. For instance, Bari and Zhang (2020) evaluated the performance of CI engines using palm oil biodiesel, reporting improved combustion efficiency and reduced emissions compared to traditional diesel fuels [16]. Similarly, Kumar et al. (2022) assessed the performance, emissions, and combustion attributes of CI engines using palm biodiesel blends and found significant improvements in engine efficiency and a reduction in harmful emissions such as NO_X_ and HC [17]. However, when TiO_2_ nanoparticles are introduced into these biodiesel blends, the improvements become even more pronounced as the catalytic activity of the nanoparticles further enhances combustion stability and reduces emissions, particularly under high engine loads.

In the case of soybean oil-based biodiesel, extensive research has also confirmed its effectiveness in reducing emissions and improving engine performance. Seraç et al. (2020) conducted a comprehensive evaluation of soybean biodiesel blends in CI engines and reported enhanced performance and lower emissions compared to conventional diesel [18]. Vellaiyan (2020) investigated the combustion, performance, and emissions of a diesel engine fueled with soybean biodiesel and its water blends, finding significant reductions in NO_X_ and particulate matter emissions [19].

A study by Hussain et al. (2020) demonstrated that the addition of 50 ppm of TiO_2_ nanoparticles to soybean biodiesel blends increased the brake thermal efficiency by 20.66% while reducing CO and HC emissions by 30% and 21.5%, respectively. However, their study also noted a slight increase in NO_X_ emissions due to the higher combustion temperatures associated with more efficient fuel oxidation [20].

Further research conducted by Lin and Lin in 2023 explored the performance of soybean biodiesel emulsions with TiO_2_ nanoparticles, revealing a significant reduction in NO_X_ and particulate matter emissions. Their experiments indicated that TiO_2_-enhanced soybean biodiesel reduced CO emissions by 25% and NO_X_ emissions by 3.75% compared to conventional diesel fuel [21]. The influence of TiO_2_ nanoparticles, combined with biodiesel’s inherent oxygen content, contributes to the reduction in unburned hydrocarbons, thus minimizing the environmental impact of biodiesel use in CI engines.

The addition of TiO_2_ nanoparticles to palm biodiesel blends has been widely studied for its potential to reduce harmful emissions in diesel engines. For example, Venu et al. (2019) demonstrated that palm biodiesel blended with TiO_2_ nanoparticles and exhaust gas recirculation (EGR) significantly reduced NO_X_ emissions, a common challenge in biodiesel combustion. The catalytic action of TiO_2_ nanoparticles improves oxidation reactions, reducing unburned hydrocarbons (HCs) and carbon monoxide (CO) emissions in palm biodiesel blends [22]. In addition, Mujtaba et al., 2020 found that the inclusion of TiO_2_ nanoparticles in palm–sesame biodiesel blends resulted in a 32.09% reduction in CO emissions and a 25.4% decrease in HC emissions compared to regular biodiesel [23]. These reductions in emissions are crucial for making biodiesel blends more viable for use in commercial diesel engines, where regulatory limits on pollutants are becoming increasingly stringent.

Furthermore, the impact of TiO_2_ nanoparticles on particulate matter (PM) emissions has been explored. Fayad et al. (2023) showed that the addition of TiO_2_ nanoparticles to biodiesel blends reduced the total concentration of particulate matter by 26.74% compared to standard diesel fuel [11]. The synergistic effect of TiO_2_ nanoparticles and EGR systems has been shown to lower NO_X_ emissions by up to 21.83% and significantly reduce PM concentrations [14]. Research indicates that TiO_2_ nanoparticles play a crucial role in improving both gaseous and particulate emissions, making palm biodiesel blends more environmentally sustainable when used in compression ignition engines.

### Identification of Research Gaps

Despite extensive research indicating the benefits of TiO_2_ nanoparticles in enhancing biodiesel properties, there remains a significant gap in understanding their specific impact on palm and soybean biofuels. Current studies often generalize the effects of TiO_2_ without delving into how these nanoparticles influence the combustion efficiency of these biofuels. Furthermore, while reductions in emissions such as NO_X_ and particulate matter have been noted, a comprehensive analysis of all emission profiles, including CO, HC, and soot particles, is lacking. This study aims to fill these gaps by providing detailed evaluations of the combustion processes and emission characteristics of CI engines fueled with TiO_2_-enhanced palm and soybean biodiesels.

In addition, while there are indications of improved engine performance metrics like power output and fuel consumption with TiO_2_-enhanced biodiesels, specific data for palm and soybean biodiesel blends are insufficient. This study seeks to systematically assess these performance metrics and explore the long-term sustainability and economic feasibility of using TiO_2_ nanoparticles in these biofuels. By addressing the stability, cost implications, and lifecycle impact of TiO_2_ integration, this research will offer a comprehensive understanding of the potential benefits and challenges, contributing valuable insights into the optimization of biofuels for sustainable engine performance.

## 3. Methodology

### 3.1. Biofuel Production

**Figure 1:**
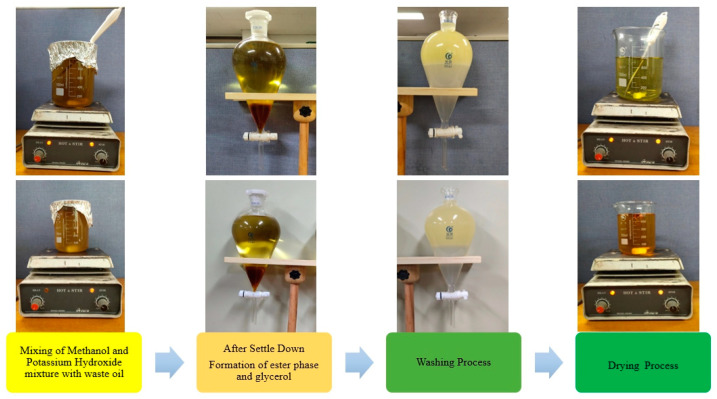
Biodiesel production: palm oil and soybean oil.

In Figure 1, the production of biodiesel from palm oil utilizing a laboratory-scale transesterification process is illustrated. The experimental setup comprised a 1000 mL flask equipped with a thermometer and a magnetic stirrer (Topscien, Ningbo, China, Model MS300) to ensure uniform mixing. Initially, 1000 mL of palm oil was heated to 65 °C in the flask. Subsequently, a solution of potassium hydroxide (12.75 g from Sigma-Aldrich, Burlington, MA, USA) dissolved in 255 mL of methanol (Sigma-Aldrich, USA, ≥99.9% purity, suitable for high-performance liquid chromatography) was added to the preheated oil. The reaction mixture was maintained under continuous stirring for 2 h. Following the reaction, the mixture was transferred to a separating funnel to separate the glycerol layer. The resulting esters were washed twice: first with warm water containing 5% acetic acid and then with plain water to purify the methyl esters. The final step involved drying the ester at 100 °C to remove any remaining alcohol and water, culminating in the production of biodiesel from palm oil.

Similarly, the synthesis of biodiesel from soybean oil was conducted using a comparable one-stage alkaline transesterification method. The apparatus included a 500 mL flask, also equipped with a thermometer and magnetic stirrer. In this instance, 500 mL of soybean oil was combined with a solution of 135 mL methanol and 2.5 g potassium hydroxide, maintaining a 10:1 molar ratio. The mixture was heated to 55 °C and stirred at a constant rate of 700 RPM for 2 h. After the reaction, the mixture was subjected to the same separation process using a funnel to remove the glycerol layer. The esters were then purified through two washes: one with warm water mixed with 5% acetic acid and the other with plain water. The final drying step at 100 °C removed any residual alcohol and water, resulting in the production of biodiesel from soybean oil.

### 3.2. Incorporation of TiO_2_ Nanoparticles

The incorporation of TiO_2_ nanoparticles into soybean oil was a carefully controlled process designed to ensure a uniform dispersion of the nanoparticles within the biodiesel blend. The TiO_2_ nanoparticles used were AEROXIDE TiO_2_ P25 (Evonik Degussa, Essen, Germany). Initially, the TiO_2_ nanoparticles were mixed with soybean oil using an ultrasonic mixer (Branson Ultrasonics, Model 2510E-MT, Danbury, CT, USA) for one hour. Ultrasonic mixing is a crucial step as it uses high-frequency sound waves to create cavitation bubbles in the liquid, which collapse and produce intense shear forces. These forces help to break apart any nanoparticle agglomerates, ensuring that the nanoparticles are evenly distributed throughout the soybean oil. This process was repeated to prevent any settling or clumping of the nanoparticles within the blend, as uniform dispersion is key to maintaining the desired properties of the fuel. Prior to testing, the nano blend was vigorously shaken and stirred for an additional two hours, further ensuring that the nanoparticles remained in suspension and did not settle. This method of incorporating TiO_2_ nanoparticles aimed to improve the fuel’s combustion characteristics and stability, potentially enhancing the overall performance of the biodiesel.

The process of incorporating TiO_2_ nanoparticles into palm oil biodiesel followed a similar methodology to that used with soybean oil, with specific adjustments made to accommodate the properties of palm oil. TiO_2_ nanoparticles, prepared through a chemical precipitation method, were first mixed into the palm oil using an ultrasonic mixer for one hour. The ultrasonic mixer facilitated the even distribution of nanoparticles by breaking down any potential clumps, thus preventing agglomeration. The mixing process was repeated to ensure the consistency and stability of the nano blend, as an uneven distribution could lead to performance inconsistencies. Additionally, to avoid settling of the nanoparticles, the mixture was subjected to vigorous shaking and stirring for two hours before any experimental testing. This step was critical in maintaining a stable dispersion as it ensured the nanoparticles were well suspended and did not settle at the bottom of the container. The incorporation of TiO_2_ nanoparticles into palm oil was carried out with the aim of enhancing the biodiesel’s properties, potentially improving the combustion efficiency, reducing emissions, and increasing fuel stability. Specific concentration levels of TiO_2_ nanoparticles (50 ppm and 75 ppm) were selected to explore their impacts on the biodiesel’s performance characteristics.

The quantities of TiO_2_ nanoparticles selected for the study—50 ppm and 75 ppm—were carefully chosen to investigate the effects of varying concentrations on the biodiesel’s performance. These specific concentrations were selected based on previous research and preliminary studies, which indicated that these levels are effective in enhancing fuel properties without causing adverse effects, such as the clogging of fuel injectors or increased viscosity:

- A concentration of 50 ppm: This moderate concentration was selected to evaluate the balance between catalytic enhancement and potential changes in fuel properties, such as viscosity and stability. It provides insights into optimal nanoparticle loading that can deliver noticeable benefits without compromising the fuel’s quality.
- A concentration of 75 ppm: The highest concentration was chosen to explore the upper limits of TiO_2_ incorporation. This level helps in understanding the maximum potential benefits and any possible drawbacks, such as increased costs or technical challenges, associated with higher nanoparticle loading.

The detailed characteristics of the TiO_2_ nanoparticles and the soybean and palm oil blends are shown in Table 1 and Table 2.

### 3.3. Experimental Setup

**Figure 2:**
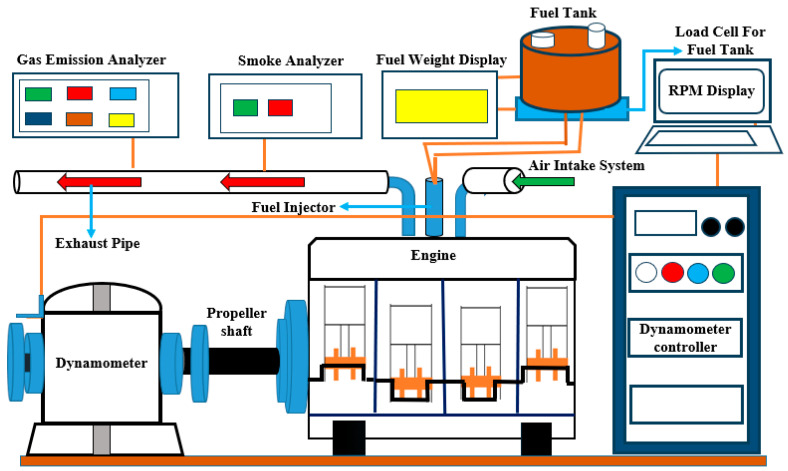
Experimental setup.

Figure 2 depicts the experimental setup used in this study, highlighting a detailed array of components essential for accurately measuring and analyzing the performance of a four-cylinder, four-stroke electronic engine. The core of the setup is the engine itself, which is connected to an eddy current dynamometer that applies varying loads to the engine. Torque measurements are precisely recorded using a load cell and a dedicated torque measurement device. Engine speed is tracked using an RPM sensor and displayed on an RPM display. Exhaust gasses are routed through an exhaust pipe and analyzed for gas composition and smoke levels using a gas emission analyzer and a smoke analyzer, respectively.

The engine receives fuel from a fuel tank, with fuel consumption being closely monitored by a load cell for fuel weight and shown on a fuel weight display. The air intake system and fuel injector work together to ensure the precise delivery of air and fuel to the engine. Additionally, a propeller shaft is included in the setup, likely serving the purpose of transmitting power from the engine. This comprehensive arrangement is meticulously designed to provide in-depth insights into the engine’s performance across various operating conditions.

### 3.4. Engine Specifications

The engine specifications detailed in Table 3 describe a four-cylinder, four-stroke power unit with electronic control, a common setup in modern vehicles that effectively balances efficiency and performance. Manufactured by Hyundai Motor Co., Ltd. in Seoul, Republic of Korea, this engine utilizes a sequential fuel injection system and follows a 1-3-4-2 firing order. This specific firing sequence helps minimize engine vibrations and enhances operational smoothness. With a displacement of 1995 cc, the engine is within the mid-range capacity for contemporary automotive engines, offering a good compromise between power and fuel efficiency. The high compression ratio of 16:1 indicates that the engine is optimized for high efficiency and may be designed to run on premium fuels. The bore and stroke are measured at 84 mm and 90 mm, respectively, suggesting a slightly over-square design that can improve engine responsiveness and potentially allow for higher RPMs. Overall, the engine’s features are tailored to provide a balanced mix of performance and efficiency, making it suitable for a wide range of driving scenarios.

This research employed a standard internal combustion engine without any modifications to ensure that the findings accurately represent the performance and emissions characteristics of engines commonly found in real-world conditions. This approach allows for a precise evaluation of different biodiesel blends alongside regular diesel, reflecting how these fuels perform in engines as they are typically configured. By avoiding any alterations to the engine, this study maintains the original design specifications, making the results highly relevant and applicable to the majority of engines currently in use. This decision ensures that the conclusions drawn from this research are both reliable and widely transferable.

### 3.5. Error Analysis and Uncertainty

During the experimental trials, the lubricating oil temperature was carefully controlled and consistently maintained within a narrow range of 85 to 90 °C. The engine was operated continuously for 15 min, during which detailed observations were made and recordings systematically documented. To minimize potential discrepancies in the data, a rigorous evaluation of uncertainty was conducted. A critical aspect of this process was the calibration of all instruments involved, ensuring the accuracy and reliability of the experimental results.

To further enhance the reliability of the findings, measurements were taken multiple times, with a minimum of four repetitions for each experiment. These repeated readings were averaged to calculate the arithmetic mean, which formed the foundation for a subsequent data analysis. This approach helped mitigate the impact of any anomalies or outliers, providing a more accurate representation of the experimental outcomes.

The detailed analysis of error and uncertainty for the smoke meter and gas analyzer is presented in Table 4. This table highlights the precision of these instruments, accounting for possible deviations and inaccuracies inherent in the measurement processes. By quantifying the uncertainty ranges and potential error margins, this table offers a clear insight into the reliability and limitations of the collected data. This comprehensive assessment is crucial for interpreting the experimental results with the necessary scientific rigor, ensuring that the conclusions drawn are both accurate and valid within the specified uncertainty bounds.

To assess the engine’s emission parameters, a CGA-4500 gas analyzer from the Republic of Korea was utilized. This sophisticated device employs Non-Dispersive Infrared (NDIR) technology to measure carbon monoxide (CO) concentrations ranging from 0.00 to 10.00% and carbon dioxide (CO_2_) levels between 0.0 and 20.0%. Additionally, it is capable of detecting hydrocarbons (HCs) within the range of 0 to 10,000 parts per million (ppm). The analyzer also features an electrochemical sensor for measuring oxygen (O_2_) levels from 0.00 to 25.00% and another electrochemical sensor specifically for quantifying nitric oxide (NO_X_) emissions, which can range from 0 to 5000 ppm.

For the acquisition of digital emission data, a probe was strategically positioned within the exhaust pipeline to ensure accurate sampling. Smoke emissions were measured using a dedicated smoke meter, providing data on the particulate content. To monitor the temperature of the exhaust gasses, a k-type thermocouple was employed. Together, these instruments allowed for a comprehensive analysis of the engine’s emission characteristics across various operational conditions, enabling a thorough evaluation of both its performance and environmental impact.

## 4. Results and Discussion

### 4.1. Emission Analysis

#### 4.1.1. Carbon Monoxide (CO) Emissions

**Figure 3:**
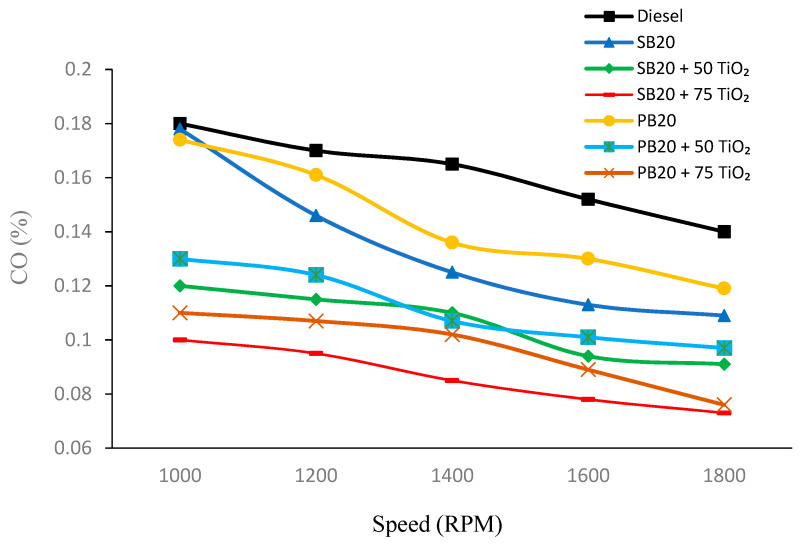
CO emission fluctuations with varying engine speed.

Figure 3 indicates that CO emissions generally decrease as the RPM increases across all fuel types. Among the fuel types, the base diesel fuel (B0) consistently shows the highest CO emissions at each RPM level, while the biofuels, particularly those with added TiO_2_ nanoparticles, exhibit lower CO emissions. For instance, at 1000 RPM, the amount of CO emissions for SB20 is 0.178%, but with the addition of 75 mg of TiO_2_, the amount of emissions reduces significantly to 0.1%. A similar trend is observed with PB20, where CO emissions drop from 0.174% to 0.11% with the addition of 75 mg of TiO_2_.

CO emissions reduced by 43.82% for SB20 with 75 mg of TiO_2_ at 1000 RPM, while PB20 showed a 36.78% reduction under similar conditions.

These findings are consistent with previous studies, which have shown that TiO_2_ nanoparticles enhance oxidative reactions, leading to a reduction in CO emissions. For instance, Jayabalaji and Shanmughasundaram in 2019 observed that incorporating TiO_2_ into biodiesel blends markedly reduced CO emissions due to the catalytic properties of the nanoparticles, which improve the combustion process [24]. Likewise, the research conducted by Gunasekar et al. (2019) confirmed that the addition of TiO_2_ nanoparticles in biodiesel blends can effectively lower CO emissions by promoting the oxidation of carbon monoxide to carbon dioxide during combustion [25].

#### 4.1.2. Hydrocarbon (HC) Emissions

**Figure 4:**
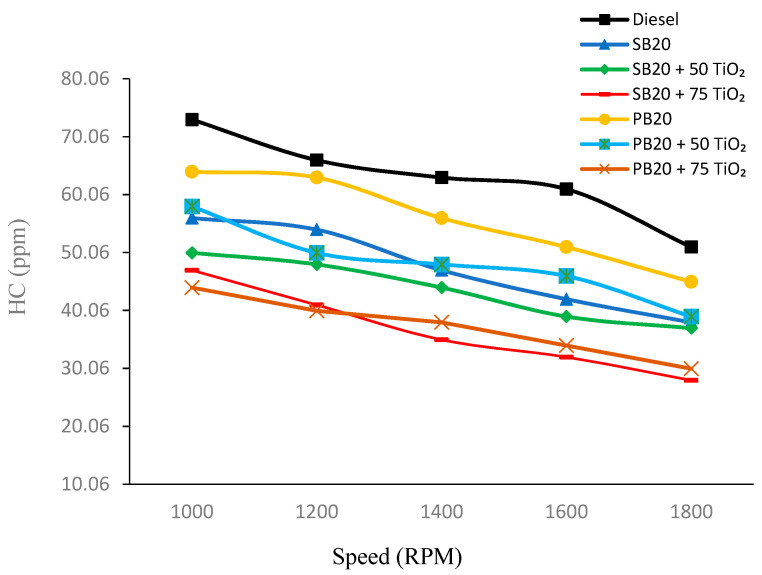
HC emission fluctuations with varying engine speed.

In Figure 4, it is evident that there is a consistent decrease in HC emissions as the RPM increases for all fuel types. Among the fuels, pure diesel (B0) exhibits the highest HC emissions at each RPM level, while the biofuels show lower HC emissions. The incorporation of TiO_2_ nanoparticles into the biofuels further reduces HC emissions, with the highest concentration of TiO_2_ (75 mg) yielding the lowest emissions. For instance, at 1000 RPM, SB20 emits 56 ppm of HC, while the SB20 + 75 TiO_2_ blend reduces this value to 47 ppm. Similarly, PB20 shows 64 ppm of HC emissions at 1000 RPM, which drops to 44 ppm with the addition of 75 mg of TiO_2_.

At the highest RPM of 1800, the trend remains clear, with SB20 + 75 TiO_2_ and PB20 + 75 TiO_2_ displaying the lowest HC emissions at 28 ppm and 30 ppm respectively. The addition of 75 mg TiO_2_ to SB20 resulted in a 16.07% reduction in HC emissions at 1000 RPM, while PB20 with 75 mg of TiO_2_ showed a 31.25% reduction. These results suggest that TiO_2_ nanoparticles significantly improve the combustion efficiency, leading to lower hydrocarbon emissions across all tested biofuels.

The reduction in HC emissions with the addition of TiO_2_ nanoparticles can be attributed to the improved oxidation and combustion efficiency provided by these nanoparticles. Nanoparticles help achieve more complete combustion, thereby reducing the amount of unburned hydrocarbons in the exhaust. The same trend was observed in previous research that demonstrated the effectiveness of TiO_2_ nanoparticles in reducing HC emissions. For instance, the research conducted by Sarma et al. (2023) showed that the incorporation of TiO_2_ nanoparticles into biodiesel blends led to a significant reduction in HC emissions due to enhanced combustion processes [26]. Similarly, Kurre et al. (2023) found that TiO_2_ nanoparticles contributed to lower HC emissions by improving the oxidation of hydrocarbons during combustion [27].

#### 4.1.3. Carbon Dioxide (CO_2_) Emissions

**Figure 5:**
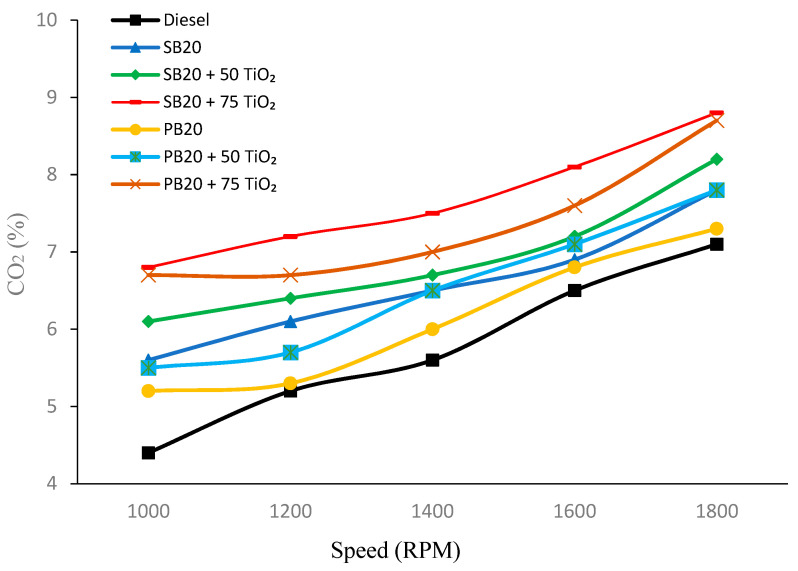
CO_2_ emission fluctuations with varying engine speed.

In Figure 5, a clear trend is observed where CO_2_ emissions increase as the RPM rises across all fuel types. Among the biofuels, the addition of TiO_2_ nanoparticles results in higher CO_2_ emissions compared to the base fuels, suggesting a more complete combustion process facilitated by the nanoparticles. For example, at 1000 RPM, SB20 emits 5.6% of CO_2_, but this increases to 6.8% with the addition of 75 mg of TiO_2_. Similarly, PB20 emits 5.2% of CO_2_ at 1000 RPM, which increases to 6.7% when 75 mg of TiO_2_ is added.

At the highest RPM of 1800, the trend is consistent, with SB20 + 75 TiO_2_ emitting 8.8% of CO_2_ compared to 4.4% for the base diesel (B0). Similarly, PB20 + 75 TiO_2_ shows 8.7% of CO_2_ emissions at 1800 RPM, highlighting the impact of TiO_2_ in enhancing the combustion efficiency, thereby increasing CO_2_ emissions due to more complete fuel oxidation.

CO_2_ emissions increase by 21.43% for SB20 and 28.85% for PB20 with 75 mg of TiO_2_ at 1000 RPM.

These findings corroborate previous research showing that TiO_2_ nanoparticles enhance combustion efficiency, leading to increased CO_2_ emissions as a greater proportion of carbon is fully oxidized during the combustion process. This outcome was observed in studies by Fangsuwannarak et al. (2020) and Madhuri et al. (2023), where the addition of TiO_2_ to biofuels resulted in higher CO_2_ emissions due to the improved catalytic combustion [28,29].

#### 4.1.4. Nitrogen Oxide (NO_X_) Emissions

**Figure 6:**
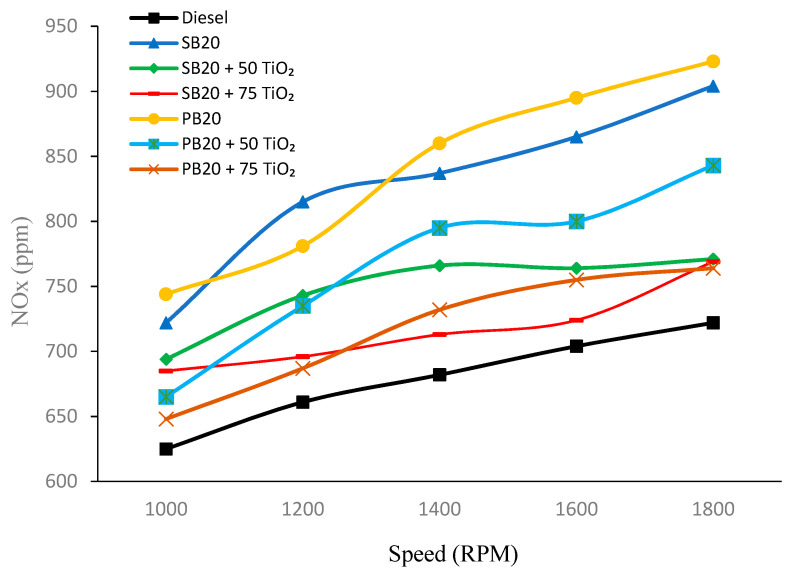
NO_X_ emission fluctuations with varying engine speed.

Figure 6 indicates that NO_X_ emissions increase with higher RPMs across all fuel types. Among the fuels, the base diesel (B0) consistently has the lowest NO_X_ emissions, while the pure SB20 biofuel exhibits the highest emissions, reaching 904 ppm at 1800 RPM. The introduction of TiO_2_ nanoparticles generally leads to a reduction in NO_X_ emissions for both SB and PB biofuels. For example, at 1000 RPM, SB20 emits 722 ppm of NO_X_, which decreases to 685 ppm when 75 mg of TiO_2_ is added.

Similarly, PB20 follows this trend, with NO_X_ emissions decreasing from 781 ppm to 687 ppm at 1200 RPM with the addition of 75 mg of TiO_2_. At higher RPMs, the reduction in NO_X_ emissions becomes more pronounced, particularly in the SB20 + 75 TiO_2_ and PB20 + 75 TiO_2_ blends, which consistently show lower emissions compared to their pure biofuel versions. The addition of TiO_2_ resulted in a 5.12% reduction in NO_X_ emissions for SB20 and 12.03% for PB20 at 1000 RPM.

These data are compared with findings from other studies in biofuel research, where the addition of TiO_2_ nanoparticles has been shown to improve combustion efficiency and reduce NO_X_ emissions due to the catalytic properties of the nanoparticles. For example, Prasetya et al. (2023) demonstrated that incorporating TiO_2_ nanoparticles in palm biodiesel blends resulted in a reduction in NO_X_ emissions due to enhanced catalytic activity during combustion [30]. Similarly, Razzaq et al. (2023) support the idea that TiO_2_ can effectively reduce NO_X_ emissions when used in biodiesel blends, particularly under high engine loads [31]. However, the data also align with the observations made by Mehregan and Moghiman in 2020, who noted that the impact of TiO_2_ nanoparticles varies significantly depending on the specific conditions, such as the type of biodiesel and engine operating parameters [32]. Thus, while TiO_2_ nanoparticles can be effective in reducing NO_X_ emissions, their efficacy is influenced by factors such as the biofuel type and engine speed.

#### 4.1.5. Exhaust Gas Temperature (EGT)

**Figure 7:**
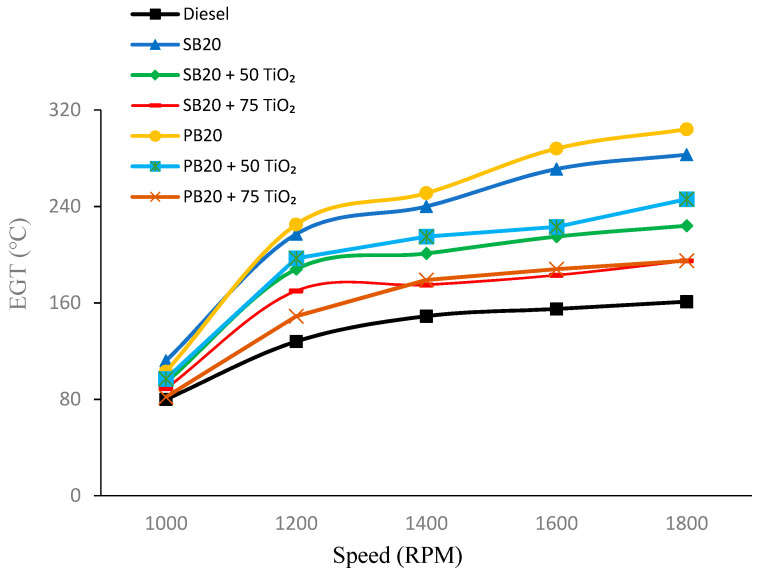
EGT fluctuations with varying engine speed.

Figure 7 reveals that the EGT increases as the RPM rises for all fuel types. Pure SB20 exhibits a consistently higher EGT compared to base diesel (B0), reaching up to 283 °C at 1800 RPM. However, when TiO_2_ nanoparticles are added, the EGT decreases for both SB and PB biofuels. For example, at 1000 RPM, the EGT for SB20 is 112 °C, but it drops to 89 °C with the addition of 75 mg of TiO_2_. Similarly, PB20 shows an EGT of 103 °C at 1000 RPM, which decreases to 82 °C when 75 mg of TiO_2_ is added.

As the engine speed increases, this trend of a reduced EGT with TiO_2_ nanoparticle addition continues. At 1800 RPM, SB20 + 75 of TiO_2_ results in an EGT of 195 °C compared to 283 °C for pure SB20. Likewise, PB20 + 75 of TiO_2_ has an EGT of 195 °C, which is significantly lower than the 304 °C temperature observed for pure PB20. The addition of TiO_2_ to SB20 at 1000 RPM resulted in a 20.54% reduction in the EGT, while PB20 showed a 25.24% reduction.

The reduction in EGT with the addition of TiO_2_ nanoparticles can be attributed to the improved combustion efficiency provided by the nanoparticles, which promotes more complete combustion and less heat being carried away by exhaust gasses. This aligns with previous research findings, where TiO_2_ nanoparticles were shown to enhance combustion characteristics, leading to lower EGTs. Studies such as those by Sarma et al. (2023) have demonstrated that the inclusion of TiO_2_ nanoparticles in biodiesel blends effectively lowers EGTs by improving combustion efficiency [26].

## 5. Conclusions

The comprehensive analysis conducted in this study underscores the significant impact of TiO_2_ nanoparticles on the emission characteristics and combustion efficiency of soybean (SB) and palm (PB) biodiesel blends in compression ignition (CI) engines. Across all test conditions, the incorporation of TiO_2_ nanoparticles consistently resulted in lower emissions of carbon monoxide (CO) and hydrocarbons (HC). This reduction is attributed to the enhanced catalytic oxidation process facilitated by TiO_2_ nanoparticles, which promotes more complete combustion. These findings are particularly noteworthy as they suggest that even at higher engine speeds, where emissions typically increase, the presence of TiO_2_ can help maintain lower pollutant levels.

In addition to reducing CO and HC emissions, this study observed an increase in carbon dioxide (CO_2_) emissions with the addition of TiO_2_ nanoparticles. While higher CO_2_ emissions might seem counterintuitive to the goals of emission reduction, they actually indicate more efficient fuel combustion as more carbon is being fully oxidized. This trade-off highlights the dual role of TiO_2_ nanoparticles in both reducing incomplete combustion by-products and ensuring more complete fuel utilization.

A critical finding from this study is the reduction in nitrogen oxide (NO_X_) emissions, which are typically challenging to control in biodiesel combustion. NO_X_ emissions generally rise with an increasing engine speed due to higher combustion temperatures. However, the addition of TiO_2_ nanoparticles demonstrated a mitigating effect, particularly at higher concentrations and RPMs, suggesting that these nanoparticles can help manage peak combustion temperatures and reduce NO_X_ formation. This effect is crucial for meeting stringent emission regulations without compromising engine performance.

By improving combustion efficiency, reducing harmful emissions, and managing exhaust temperatures, these nanoparticles offer a promising pathway toward more sustainable and efficient biodiesel usage in diesel engines. Future research could explore the long-term effects of TiO_2_ nanoparticle use on engine wear and the potential for further optimizing nanoparticle concentrations to balance emissions and performance. The insights gained from this study can inform the development of next-generation biodiesel formulations that align with global efforts to reduce carbon footprints and promote cleaner energy solutions in the transportation sector.

## 6. Future Scope

While this study offers important insights into the effects of TiO_2_ nanoparticles on biofuel emissions and performance, several areas merit further research:

- The Fate of TiO_2_ Nanoparticles: Future studies should investigate whether TiO_2_ nanoparticles remain in the soot, are filtered, or are emitted into the atmosphere post-combustion.
- Fuel Consumption: Future research should evaluate the impact of TiO_2_ on fuel economy, which was not assessed in this study.
- Smoke and Particulate Emissions: Measuring smoke opacity and particulate matter emissions will provide a more comprehensive understanding of TiO_2_’s environmental impact.
- Stability, Costs, and Life Cycle Impact: Studies should assess the long-term stability, cost-effectiveness, and environmental life cycle of TiO_2_-enhanced biofuels.
- Nanoparticle Dispersion: While ultrasonic mixing was used to disperse TiO_2_, future work should employ techniques like SEM or TEM to verify nanoparticle distribution and long-term stability in biofuels.
- Sample Testing: Testing each fuel sample three times would improve statistical reliability. Future studies should aim to carry out triplicate testing to enhance data robustness.
- The Uniformity of TiO_2_ Dispersion: Variations in viscosity and calorific value may result from slight inconsistencies in nanoparticle dispersion. Future work will focus on advanced testing to ensure better uniformity in the samples.

These areas provide significant opportunities to further optimize the integration of TiO_2_ nanoparticles into biofuels for improved engine performance and environmental sustainability.

## Figures and Tables

**Table 1:** Nanoparticle (TiO_2_) properties.

Detail	Properties
Name (TiO_2_)	Nanoparticles of titanium dioxide
Appearance	White
Purity of TiO_2_	96%
Particle size	20–50 nm
Surface area	>42 m^2^/g
Melting point	>233 °C

**Table 2:** Fuel characteristics.

Fuel Type	Density (kg/m^3^)	Viscosity (cSt)	Flash Point (°C)	Cetane Number	Calorific Value (MJ/kg)	Ester Content (%)
Diesel	0.820	2.87	58	48.7	45.515	-
SB20	0.834	2.92	79	52	43.536	96.2
SB20 + 50 ppm TiO_2_	0.841	3.19	86	53.5	43.607	96.5
SB20 + 75 ppm TiO_2_	0.846	3.27	89	53.8	43.695	96.8
PB20	0.862	3.10	82	51.5	43.201	97.1
PB20 + 50 ppm TiO_2_	0.868	3.36	88	52.9	43.272	97.4
PB20 + 75 ppm TiO_2_	0.872	3.45	91	53.2	43.335	97.7

**Table 3:** Engine specification and data points.

Parameters	Specifications
Engine Type	Electronic, four-cylinder, four-stroke type
Injection Sequence	1-3-4-2
Stroke Length (mm)	90
Bore (mm)	84
Compression Ratio	16:1
Displacement (cc)	1995

**Table 4:** Measuring range and precision of smoke meter and gas analyzer.

Exhaust Emission	Range	Resolution	Accuracy and Uncertainties
CO	0.00–10.00	%	±0.001%
HC	0–10,000	ppm	±1 ppm
CO_2_	0.0–20.0	%	±0.01%
O_2_	0.00–25.00	%	±0.01%
NO_X_	0–5000	ppm	±1 ppm
Smoke	0–100	%	±0.05%
Thermocouple (K-Type)	0–1200	℃	±0.1 °C

## Data Availability

Data are contained within the article.

## Abbreviations

kg/m^3^	Kilograms per cubic meter (density)
cSt	Centistokes (viscosity)
°C	Degrees Celsius (flash point)
ppm	Parts per million (nanoparticle concentration)
MJ/kg	Megajoules per kilogram (calorific value)
%	Percentage
NO_X_	Nitrogen oxide
CO	Carbon monoxide
HC	Hydrocarbon
CO_2_	Carbon dioxide
EGT	Exhaust gas temperature
CI Engine	Compression ignition engine
TiO_2_	Titanium dioxide
SB20	20% soybean biodiesel and 80% diesel blend
SB20 + 50 ppm TiO_2_	SB20 blend with 50 ppm TiO_2_ nanoparticles
SB20 + 75 ppm TiO_2_	SB20 blend with 75 ppm TiO_2_ nanoparticles
PB20	20% palm biodiesel and 80% diesel blend
PB20 + 50 ppm TiO_2_	PB20 blend with 50 ppm TiO_2_ nanoparticles
PB20 + 75 ppm TiO_2_	PB20 blend with 75 ppm TiO_2_ nanoparticles
